# Two Distinct *Triatoma dimidiata* (Latreille, 1811) Taxa Are Found in Sympatry in Guatemala and Mexico

**DOI:** 10.1371/journal.pntd.0000393

**Published:** 2009-03-10

**Authors:** Patricia L. Dorn, Claudia Calderon, Sergio Melgar, Barbara Moguel, Elizabeth Solorzano, Eric Dumonteil, Antonieta Rodas, Nick de la Rua, Roberto Garnica, Carlota Monroy

**Affiliations:** 1 Loyola University New Orleans, New Orleans, Louisiana, United States of America; 2 University of Wisconsin, Madison, Wisconsin, United States of America; 3 University of San Carlos, Ciudad Universitaria, Guatemala; 4 Universidad Autónoma de Yucatán, Merida, Yucatan, Mexico; Universidad de Los Andes, Colombia

## Abstract

Approximately 10 million people are infected with *Trypanosoma cruzi*, the causative agent of Chagas disease, which remains the most serious parasitic disease in the Americas. Most people are infected via triatomine vectors. Transmission has been largely halted in South America in areas with predominantly domestic vectors. However, one of the main Chagas vectors in Mesoamerica, *Triatoma dimidiata*, poses special challenges to control due to its diversity across its large geographic range (from Mexico into northern South America), and peridomestic and sylvatic populations that repopulate houses following pesticide treatment. Recent evidence suggests *T. dimidiata* may be a complex of species, perhaps including cryptic species; taxonomic ambiguity which confounds control. The nuclear sequence of the internal transcribed spacer 2 (ITS2) of the ribosomal DNA and the mitochondrial cytochrome b (mt *cyt b*) gene were used to analyze the taxonomy of *T. dimidiata* from southern Mexico throughout Central America. ITS2 sequence divides *T. dimidiata* into four taxa. The first three are found mostly localized to specific geographic regions with some overlap: (1) southern Mexico and Guatemala (Group 2); (2) Guatemala, Honduras, El Salvador, Nicaragua, and Costa Rica (Group 1A); (3) and Panama (Group 1B). We extend ITS2 Group 1A south into Costa Rica, Group 2 into southern Guatemala and show the first information on isolates in Belize, identifying Groups 2 and 3 in that country. The fourth group (Group 3), a potential cryptic species, is dispersed across parts of Mexico, Guatemala, and Belize. We show it exists in sympatry with other groups in Peten, Guatemala, and Yucatan, Mexico. Mitochondrial *cyt b* data supports this putative cryptic species in sympatry with others. However, unlike the clear distinction of the remaining groups by ITS2, the remaining groups are not separated by mt *cyt b*. This work contributes to an understanding of the taxonomy and population subdivision of *T. dimidiata*, essential for designing effective control strategies.

## Author Summary

The Chagas disease parasite, transmitted to humans by triatomine bugs, remains a leading cause of heart and digestive disease in Latin America. Pesticide spraying has effectively halted transmission in most of southern South America, especially where the bugs live exclusively inside houses. In Mesoamerica, bugs living in the forest readily reinfest treated houses. In addition, one of the main species of insect that transmits Chagas in Mesoamerica, *Triatoma dimidiata*, although it looks similar in different localities, may consist of genetically distinct populations, even different species, which differ in how efficiently they transmit the parasite: characteristics which confound control efforts. Nuclear and mitochondrial DNA were analyzed to characterize different populations of *T. dimidiata* from Mexico and Central America. Both the nuclear and mitochondrial DNA show that there is a very distinct population of *T. dimidiata*, perhaps even a different species, that lives in very close proximity with other *T. dimidiata* in Mexico and Guatemala. The nuclear DNA divides the remaining *T. dimidiata* into three additional genetically distinct groups. However, the mitochondrial DNA does not distinguish these additional groups. This study helps inform control efforts by showing where genetically distinct populations of *T. dimidiata* occur.

## Introduction

### Importance of Chagas

Chagas disease is considered the largest parasitic disease burden in Latin America with a cost of the loss of 667,000 Disability Adjusted Life Years (DALYs) in 2002 [1]. *Trypanosoma cruzi*, the parasite that causes Chagas disease, infects approximately 9.8 million people in the Americas [2] with 200,000 new Chagas cases annually [3]. Thus, Chagas disease remains a serious health problem in the Americas. Most transmission occurs by contamination with the parasite-containing feces of triatomine insect vectors (Hemiptera:Reduviidae). There is no vaccine available and treatment shows limited effectiveness, comes with troublesome side effects, and is out of reach of most people in endemic countries. Therefore, as with most parasitic infections, control of transmission by the vectors is the control strategy of choice.

### Control

A greater than 94% reduction in Chagas transmission has been realized in South America through the Southern Cone Initiative, a concerted effort of the Ministries of Health and the World Health Organization [4]. This initiative is focused on reduction in vector populations by residual pesticide application in houses and mandatory blood screening. The fact that the targeted S. American vectors are almost exclusively domestic (remain in houses) has greatly facilitated the control efforts. In fact, in regions with significant peridomestic and sylvan vector populations (e.g. the Gran Chaco region), control is still a challenge [5]. In Central America, Mexico, and regions of S. America, many vectors occupy sylvan and peridomestic as well as domestic habitats which poses serious challenges to control efforts as these extra-domiciliary sites serve as reservoirs to repopulate treated houses.

### The Triatominae

The Triatominae comprises 141 species grouped into 18 genera forming six tribes ([6],[7] and references therein) and more than half of these are naturally infected with *T. cruzi* [8]. The most important genera involved in Chagas transmission include: *Triatoma*, *Rhodnius* and *Panstrongylus* and epidemiologically significant species within these genera have been described for most endemic regions [8]. The risk particular species pose for transmission to humans is affected by several aspects of triatomine biology and behavior such as: food preference and frequency of feeding, infestation and crowding indices, likelihood and conditions for dispersal, fecundity, and especially the degree of adaptation to the domestic environment which puts them in close contact with human hosts. As humans move into infested sylvan areas, vectors appear to be able to adapt to human dwellings and some species have evolved towards domesticity [9]. An unambiguous identification of vector species and an understanding of the divisions among taxa in endemic areas are critical to an understanding of the epidemiology of the disease and to its control.

### Taxonomy of *Triatoma dimidiata* is unclear

Considerable morphological variation of *Triatoma dimidiata* (Latreille, 1811), the most important Chagas vector in Central America [10], historically has led to splitting, merging and name changes of the species (reviewed in [11]). *T. dimidiata* is found from Mexico, throughout Central America, and in northern S. America including: Colombia, Ecuador and into to northern Peru. First two separate species were described: *Conorhinus dimidiatus* from Ecuador, Costa Rica, and Panama; and *C. maculipennis* from Mexico [12] then quickly synonymized [13], as has occurred again more recently under its current name, *T. dimidiata*, considering that the variation was “roughly clinal in nature” [14]. However, its taxonomy remains problematic as results of recent morphological evaluation have led taxonomists to assert that *T. dimidiata* is more aptly considered a species complex [15]. Analysis of antenna sensilla [16], head morphometry [17], cuticular hydrocarbon patterns [18] suggested *T. dimidiata* was divided into two, three or four taxa, respectively, dividing roughly between Southern Mexico and Guatemala, often with population outliers, such as the Lanquin cave population in Guatemala and the Yucatan, Mexico population (sometimes, but not always, grouped with nearby Peten, Guatemala). Cytogenetic analysis shows three distinct “cytotypes”, however, the divisions differ from those described using phenotypic markers. For example, cytogenetics distinguishes isolates from Peten, Guatemala and Yucatan, Mexico from each other and all other isolates [19], however, other markers, such as cuticular hydrocarbon patterns, show them clustering [18].

### Molecular tools contribute to resolving questionable taxonomies

Molecular tools are increasingly being used to clarify relationships at all taxonomic levels including tribes, genera, species, subspecies and even populations. The internal transcribed spacer 2 (ITS2) has been particularly useful for analyzing populations of arthropod vectors. ITS2 is part of the rDNA cistron found between the 5.8S and 28S rDNA and is present in hundreds of tandemly repeated copies in the eukaryotic genome. Since the role of ITS2 is to assist with the processing of the 45S precursor RNA to rRNA subunits, only sequences required for its secondary structure need to be conserved [20]. Its high rate of mutation has made it useful for distinguishing species [21],[22], uncovering cryptic species [23], and importantly, identifying species responsible for human infection [24]. Assays based on ITS2 can then be developed to identify species [23]. It is often necessary to include several molecular markers to unambiguously resolve taxonomies [25]. The mitochondrial gene, cytochrome b (mt *cyt b*), codes for a protein involved in the electron transport chain. Since it is mitochondrial (as opposed to the nuclear ITS2), and protein coding, it provides a distinct marker for taxa subdivision.

### Molecular tools and *T. dimidiata*

Within the Triatominae, ITS2 has become increasingly important [26] and been used to identify two major clades in the Triatomini; one consisting of North and Central American species and the other South American [27], to demonstrate that certain populations were introduced from elsewhere [27],[28], and challenge previous taxonomic arrangements [29].

Among the Triatomini, mt *cyt b* has been used to understand phylogenetic relationships and population genetic structure, challenge taxonomic status, infer ancestral populations and source of reinfesting populations [30]–[36]. Mitochondrial *cyt b* has been used to understand divisions among triatomine complexes, but has not yet been used to determine *T. dimidiata* taxa.

The clinal variation among *T. dimidiata* populations, suggested by Lent and Wygodzinsky [14] was supported by a study of the male external genitalia on a limited number of samples [37]. Clinal variation was also initially supported by ITS2 studies showing Southern Mexican populations nearly indistinguishable (Yucatan excluded) but increasing differences when compared to Central American populations (Nicaragua and Honduras) [27]. Preliminary ITS2 data showed three distinct taxa, rather than clinal variation; the divisions were: (1) southern Mexico, (2) Central America and (3) Yucatan, Mexico grouped with Peten, Guatemala, this latter group as separate as a different species [11]. In fact, cryptic species may exist in the Yucatan [27],[38]. An “outlier species” is also suggested by cuticular hydrocarbon patterns [18] and cytogenetics [19]. However, these “outliers” are sometimes found in different geographic regions from the putative cryptic species and since different analyses were done on different specimens, it is impossible to tell if they are identifying the same putative cryptic species. Recently, additional ITS2 analyses have shown that two distinct taxa exist in another state in the Yucatan peninsula, Campeche, Mexico, (one taxon includes samples from Central America); the taxa occupying different geographic regions and habitats [39]. And very recently, in isolates from: Mexico, Guatemala, Honduras, Nicaragua, Panama, Colombia and Ecuador, 31 *T. dimidiata* ITS2 haplotypes were identified falling in four distinct groups, referred to as groups 1A and B, 2 and 3, including one that is proposed to be a separate species (Group 3, *T.* sp. aff. *dimidiata*) [38]. This proposed cryptic species (Group 3) was found in Chiapas and Yucatan, Mexico; Peten, Guatemala; and Yoro, Honduras. So it is clear that the most diversity of *T. dimidiata* is found in the region encompassing southern Mexico through northern Guatemala (perhaps as far south as Honduras) and extending east through the Yucatan peninsula and this is the region where both *T. dimidiata* (or subspecies of *T. dimidiata*) and *T.* sp. aff. *dimidiata* (Group 3, proposed cryptic species) occur. Nothing is yet known about populations in Belize. We analyzed 53 *T. dimidiata* samples across this most diverse region and Belize by ITS2 and partial mt *cyt b* sequences from a subset of these samples to further understand the taxonomic subdivisions of *T. dimidiata* among Mesoamerican populations.

Knowing the clear identity of the vector species, the dividing lines between different populations and the mechanisms maintaining these divisions is critical to effective control of transmission of Chagas disease [40]. Identification of genetically similar populations could suggest shared characteristics such as: food and habitat preference, tendencies towards domestication, feeding and mating behavior, time and conditions of dispersal, fecundity, etc. Many of these characteristics are directly related to vector competence. The degree of subdivision will indicate the risk of repopulation from nearby populations following control and the degree of genetic variation within a population can suggest the risk of acquisition of insecticide resistance. Genetic markers can also identify the source of re-infesting insects [41]. An understanding of the mechanism of population subdivision may lead to novel control strategies.

## Materials and Methods

### Specimen collection and DNA isolation

The sample information for the *T. dimidiata* specimens studied with ITS2 are shown in Table 1. *Triatoma* samples used for the study of mt *cyt b* sequence are given in Table 2. All *T. dimidiata* were identified using the key of Lent and Wygodzinsky [14]. Bugs were collected during 2000–2007 by trained personnel using the person-hour collection method or in the case of some Yucatan, Mexico and Belize samples, by householder collection. All samples were collected inside houses (domestic), except those indicated as peridomestic (collected in outbuildings, woodpiles, etc. nearby the house), or sylvan (forest, Table 1). The bug's legs were removed and stored at −4°C in 95% alcohol with 5% glycerol until DNA isolation.

**Table 1:** *T. dimidiata* samples studied, including ITS2 group, haplotype, and sequence length in base pairs.

No.	Collection site	ITS2 group	ITS2 haplotype	Ecotope	ITS2 length	Genbank accession No.
1	Mérida, Yucatán, **Mexico**	2	T.dim-H32 *	domestic	495	FJ197146
2	Mérida, Yucatán, **Mexico**	3	T.dim-H36*	domestic	494	FJ197150
3	Mérida, Yucatán, **Mexico**	3	T.dim-H33 *	domestic	494	FJ197147
4	Túxpan, Veracruz, **Mexico**	1B	T.dim-H34*	domestic	496	FJ197148
5	Túxpan, Veracruz, **Mexico**	2	T.dim-H18	domestic	496	DQ871354
6	Yaxhá, Petén, **Guatemala**	3	T.dim-H35*	sylvan	494	FJ197149
7	Yaxhá, Petén, **Guatemala**	2	T.dim-H18	sylvan	496	DQ871354
8	Yaxhá, Petén, **Guatemala**	2	T.dim-H18	sylvan	496	DQ871354
9	Yaxhá, Petén, **Guatemala**	3	T.dim-H36*	sylvan	494	FJ197150
10	Yaxhá, Petén, **Guatemala**	3	T.dim-H36*	sylvan	494	FJ197150
11	Los Amates, Izabal, **Guatemala**	1A	T.dim-H01	sylvan	497	AM286693
12	Los Amates, Izabal, **Guatemala**	1A	T.dim-H03	sylvan	497	AM286695
13	Cobán, Alta Verapaz, **Guatemala**	1A	T.dim-H01	domestic	497	AM286693
14	La Brea, Quezada, Jutiapa, **Guatemala**	1A	T.dim-H01	domestic	497	AM286693
15	La Brea, Quezada, Jutiapa, **Guatemala**	1A	T.dim-H01	domestic	497	AM286693
16	La Brea, Quezada, Jutiapa, **Guatemala**	1A	T.dim-H01	domestic	497	AM286693
17	La Brea, Quezada, Jutiapa, **Guatemala**	1A	T.dim-H01	domestic	497	AM286693
18	La Brea, Quezada, Jutiapa, **Guatemala**	1A	T.dim-H04	domestic	497	AM286696
19	Valle Abajo, Acatempa, Jutiapa, **Guatemala**	1A	T.dim-H01	domestic	497	AM286693
20	Valle Abajo, Acatempa, Jutiapa, **Guatemala**	1A	T.dim-H03	domestic	497	AM286695
21	Xepatzac, Sajcabaja, Quiché, **Guatemala**	2	T.dim-H18	domestic	496	DQ871354
22	Aldea Lililla, San Andrés Sajcabajá, Quiché, **Guatemala**	2	T.dim-H18	domestic	496	DQ871354
23	San Andrés Sajcabajá, Quiché, **Guatemala**	2	T.dim-H37*	domestic	496	FJ197151
24	Chaoj, Sacapulas, Quiché, **Guatemala**	2	T.dim-H18	domestic	496	DQ871354
25	Chaoj, Sacapulas, Quiché, **Guatemala**	2	T.dim-H18	domestic	496	DQ871354
26	Lanquin, Alta Verapaz, **Guatemala**	1A	T.dim-H10	sylvan	496	AM286702
27	Lanquin, Alta Verapaz, **Guatemala**	1A	T.dim-H10	sylvan	496	AM286702
28	Lanquin, Alta Verapaz, **Guatemala**	1A	T.dim-H10	sylvan	496	AM286702
29	Rabinal, Baja Verapaz, **Guatemala**	2	T.dim-H18	domestic	496	DQ871354
30	Rabinal, Baja Verapaz, **Guatemala**	2	T.dim-H18	domestic	496	DQ871354
31	Pueblo Nuevo Viñas, St.a Rosa, **Guatemala**	1A	T.dim-H01	domestic	497	AM286693
32	Sta. Theresa, Toledo district, **Belize**	2	T.dim-H18	domestic	496	DQ871354
33	San Pedro Columbia, Toledo district, **Belize**	2	T.dim-H39*	domestic	499	FJ197153
34	Calla Creek, Cayo district, **Belize**	3	T.dim-H38*	domestic	496	FJ197152
35	El Lodo Negro, San Marcos, Sierra Intibuca, **Honduras**	1A	T.dim-H02	domestic	496	AM286694
36	Sta. Rosa de Copán, Copán, **Honduras**	1A	T.dim-H01	domestic	497	AM286694
37	El Masical, San Antonio, Copán, **Honduras**	1A	T.dim-H02	domestic	496	AM286694
38	Yoro, **Honduras**	1A	T.dim-H02	domestic	496	AM286694
39	Yoro, **Honduras**	1A	T.dim-H02	domestic	496	AM286694
40	Santa Ana, **El Salvador**	1A	T.dim-H04	domestic	497	DQ871355
41	Santa Ana, **El Salvador**	1A	T.dim-H04	domestic	497	DQ871355
42	El Peligro, El Almendro, Rio San Juan, **Nicaragua**	1A	T.dim-H01	domestic	497	AM286693
43	Villa Alvarez, El Almendro, Río San Juan, **Nicaragua**	1A	T.dim-H01	domestic	497	AM286693
44	Jinotepe, Carazo, **Nicaragua**	1A	T.dim-H01	domestic	497	AM286693
45	Jinotepe, Carazo, **Nicaragua**	1A	T.dim-H01	domestic	497	AM286693
46	Masatepe, Masaya, **Nicaragua**	1A	T.dim-H01	domestic	497	AM286693
47	Matagalpa, Escipulas, **Nicaragua**	1A	T.dim-H01	domestic	497	AM288693
48	Sto. Tomás, Sto. Domingo, Heredia, **Costa Rica**	1A	T.dim-H01	peridomestic	497	AM286693
49	Getsemani, Angeles, San Rafael, Heredia, **Costa Rica**	1A	T.dim-H01	peridomestic	497	AM286693
50	Getsemani, Angeles, San Rafael, Heredia, **Costa Rica**	1A	T.dim-H01	peridomestic	497	AM296693
51	Santa Fe, Veraguas, **Panamá**	1B	T.dim-H16	domestic	497	AM28608
52	Santa Fe, Veraguas, **Panamá**	1B	T.dim-H16	domestic	497	AM28608
53	Santa Fe, Veraguas, **Panamá**	1B	T.dim-H16	domestic	497	AM28608

**Table 2:** Samples used for mt *cyt b* sequences analysis.

Country	Population ID (Sample No.)	Collection site	Genbank accession No.
Mexico	MxHd01	Hidalgo	AY062151
Mexico	MxYuMe01 (1)	Mérida	FJ197157
Mexico	MxYuMe02 (2)	Mérida	FJ197158
Mexico	MxYuMe03 (3)	Mérida	FJ197159
Mexico	MxVr01	Veracruz	AY062149
Mexico	MxVr02	Veracruz	AY062150
Mexico	MxYu03	Yucatán	AY062163
Mexico	MxYu04	Yucatán	AY062162
Mexico	MxYu05	Yucatán	AY062160
Mexico	MxYu06	Yucatán	AY062161
Mexico	MxYu07	Yucatán	AY062159
Mexico	MxYu02	Yucatán	AY062158
Mexico	MxYu01	Yucatán	AY062164
Guatemala	GtSR01	Santa Rosa	AY062157
Guatemala	Gt0002	Unknown	AF301594
Belize	BzToST32 (32)	Santa Theresa, Toledo district	FJ197155
Belize	BzToSP33 (33)	San Pedro Columbia, Toledo district	FJ197154
Belize	BzCaCC34 (34)	Calla Creek, Cayo district	FJ197156
Honduras	HnTg01	Tegucigalpa	AY062156
Honduras	HnTg02	Tegucigalpa	AY062154
Honduras	HnTg03	Tegucigalpa	AY062152
Honduras	HnTg04	Tegucigalpa	AY062155
Honduras	HnYo01	Yoro	AY062153
Mexico	*T. pallidipennis*	Unknown	DQ198814

Mérida, MX, and Belize sample number refers to Table 1. All samples are *T. dimidiata* unless noted.

DNA was isolated from two (adults) or three (nymphs) bug legs exactly as described in Dorn et al. [42] using the method originally from [43] with modifications as described in [44] and below. Briefly, bug legs from individual bugs were separately ground using a Kontes pestle in 100 µl grind buffer (0.1 M NaCl, 0.2 M sucrose, 50 mM EDTA, 100 mM Tris-HCl [pH 8.0–9.0], 0.05%SDS). The debris was removed by centrifuging the lysate briefly at 14,000×g. The homogenate was incubated at 65°C for 15–30 min. 8 M potassium acetate was added for a final concentration of 1 M potassium acetate and the solution incubated for 15 minutes on ice to precipitate the SDS. The sample was centrifuged at 14,000×g at 4°C for 10 minutes and the supernatant transferred to a cold 1.5 ml microfuge tube. 2.5× volumes of 100% ethanol were added to precipitate the DNA. The sample was then incubated on ice for at least 10 minutes and centrifuged for 20 min at 14,000×g at 4°C. The resultant pellet was washed with 70% ethanol, allowed to dry and then resuspended in 50 µl sterile TE buffer (10 mM Tris-Cl, pH 7.5, 1 mM EDTA, pH 8.0) containing 1 U RNAase A (Sigma-Aldrich Co., St. Louis, MO). The samples were then stored at −80°C until amplification. Several DNA samples were left at room temperature for ∼1 month due to power failure following Hurricane Katrina. Only those that still amplified following this treatment were used in the analysis.

### PCR, sequencing and alignment

The ITS2 region was amplified from 3% of the isolated DNA in a 50 µl reaction (3.5 mM MgCl_2_, 2 U *Taq* DNA polymerase), exactly according to manufacturer's instructions (Applied Biosystems, Foster City, CA) using primers that anneal to the conserved 5.8S and 28S rDNA that flank the ITS2 region [27]: 5′-CTAAGCGGTGGATCACTCGG-3′ (5,8T) and 5′-GCACTATCAAGCAACACGACTC-3′ (28T). Amplification conditions were as follows: one cycle at 94°C 2 min; followed by 30 cycles of: 94°C - 30 sec, 58°C - 30 sec, 72°C - 30 sec and a final polishing step of 72°C for 7 min. The mitochondrial cytochrome b gene (mt *cyt b*) was amplified using primers CYTB7432F, 5′-GGACG(AT)GG(AT)ATTTATTATGGATC, and CYTB7433R, 5′GC(AT)CCAATTCA(AG)GTTA(AG)TAA [45] using amplification conditions: one cycle at 94°C 3 min; followed by 30 cycles of: 94°C – 1 min, 45°C – 1 min, 72°C – 1 min and a final polishing step of 72°C for 10 min. Ten percent of the amplified product was visualized by Agarose Gel Electrophoresis and UV transillumination and successfully amplified products purified using QIAquick PCR purification kit or the QIAquick Gel Extraction Kit (QIAGEN, Valencia, CA). Both strands were completely sequenced and sequences edited using MacVector software (version 9.5, Accelrys, San Diego, CA) or Bioedit version 7.0.9 [46] and ClustalW version 2.0 [47] was used to align the data from within Bioedit or MacVector. The Staden Package (version 1.6.0) [48],[49] was used to obtain the haplotype sequences and group identical sequences. DnaSP v 4.50.3 was used to find the polymorphic and parsimony informative sites in the haplotypes [50].

The average evolutionary distance within and between the groups was determined by a Kimura 2-parameter distance calculation (MacVector 10.0), based on the assumption that all sites evolve at the same rate and counting only substitutions, indels are excluded [51]. The estimated time of divergence (below diagonal, Table 3) was calculated using the Kimura 2-parameter distance and the base substitution rate, r = 44.1–99.4 X ^−10^ per site per year calculated by Bargues et al. [52] for ITS2 in Triatomini.

**Table 3:** Kimura Two Parameter distance within (diagonal, bold type) or among ITS2 groups (above diagonal) and time separated (below diagonal).

Group	1A	1B	2	3
**1A**	**0.0043**	0.0081	0.0203	0.0322
**1B**	.407–.985 mya	**0.0035**	0.0151	0.0323
**2**	1.02–2.47 mya	.76–1.84 mya	**0.0014**	0.0429
**3**	1.62–3.92 mya	1.62–3.93 mya	2.16–5.22 mya	**0.0032**

Hierarchical partitioning of molecular variance was tested using AMOVA [53] in Arlequin ver. 3.0 [54].

### Taxon sampling and phylogenetic analysis

Maximum Parsimony (MP) analysis was conducted on all characters equally weighted both including and excluding gap characters. Heuristic searches were conducted in PAUP*, version 4.0b10 [55] using 1,000 random taxon addition replicates, holding one single tree in each step and using TBR (Tree Bisection and Reconnection), a branch swapping algorithm for tree search. To estimate clade support 1,000 bootstrap replicates were subject to heuristic searches using 1,000 random taxon additions and TBR branch swapping, giving 463 constant characters, 26 parsimony-informative variable characters.

ML analysis was conducted only on DNA characters, heuristic searches using 1000 random addition replicates and TBR branch swapping were completed under the best fit model (K81uf+I+G), selected by Akaike Information Criterion (AIC) implemented in Modeltest 3.7. The parameters used were: A(0.3191), C(0.1063), G(0.1371), T(0.4375), Nst = 6, Rmat = (1.0000 9.7678 3.4276 3.4276 9.7678) Rates = gamma Shape = 0.9635 Pinvar = 0.7352 [55].

Bayesian phylogenetic analysis was conducted in MrBayes version 3.1 [56]. The sequences were analyzed under the K81uf+I+G model. Clade support was estimated using a Markov Chain Monte Carlo (MCMC) algorithm [56], set to analyze 8 linked chains (sequential heat = 0.1) with four independent runs for 2,000,000 generations sampling every 100 generations. Stability of the process was assessed by plotting the likelihood scores against generation time and 25% of the trees were discarded as part of the burn in.

The full-length ITS2 [38] and mt *cyt b* sequences [57] (Harris, KD and Beard, CB) available on GenBank are included for comparison. The sequence of *T. pallidipennis* was used as an outgroup [34],[38] (Accession no. for ITS2, AJ286882).

Median-joining network analysis was performed using Network (version 4.5.0.0, Fluxus Technology, Suffolk, England; fluxus-engineering.com) [58].

## Results

### Sequence analysis

The portion of the DNA containing the ribosomal ITS2 sequence was amplified from 53 specimens of *T. dimidiata* using primers to the 5.8S and 28S rDNA resulting in approximately 900 bp fragments. The ITS2 region was identified according to Bargues et al. [38] and ranged from 489–499 bp in *T. dimidiata* (Table 1). The sequences were strongly A+T biased at 75–76%. DNA for amplification of the mt *cyt b* gene was available from a subset of six samples (three each from Belize and Yucatan, Mexico) and trimmed to 665 nt for comparison with sequences available on GenBank (Table 2).

### ITS2 haplotypes

**Figure 1:**
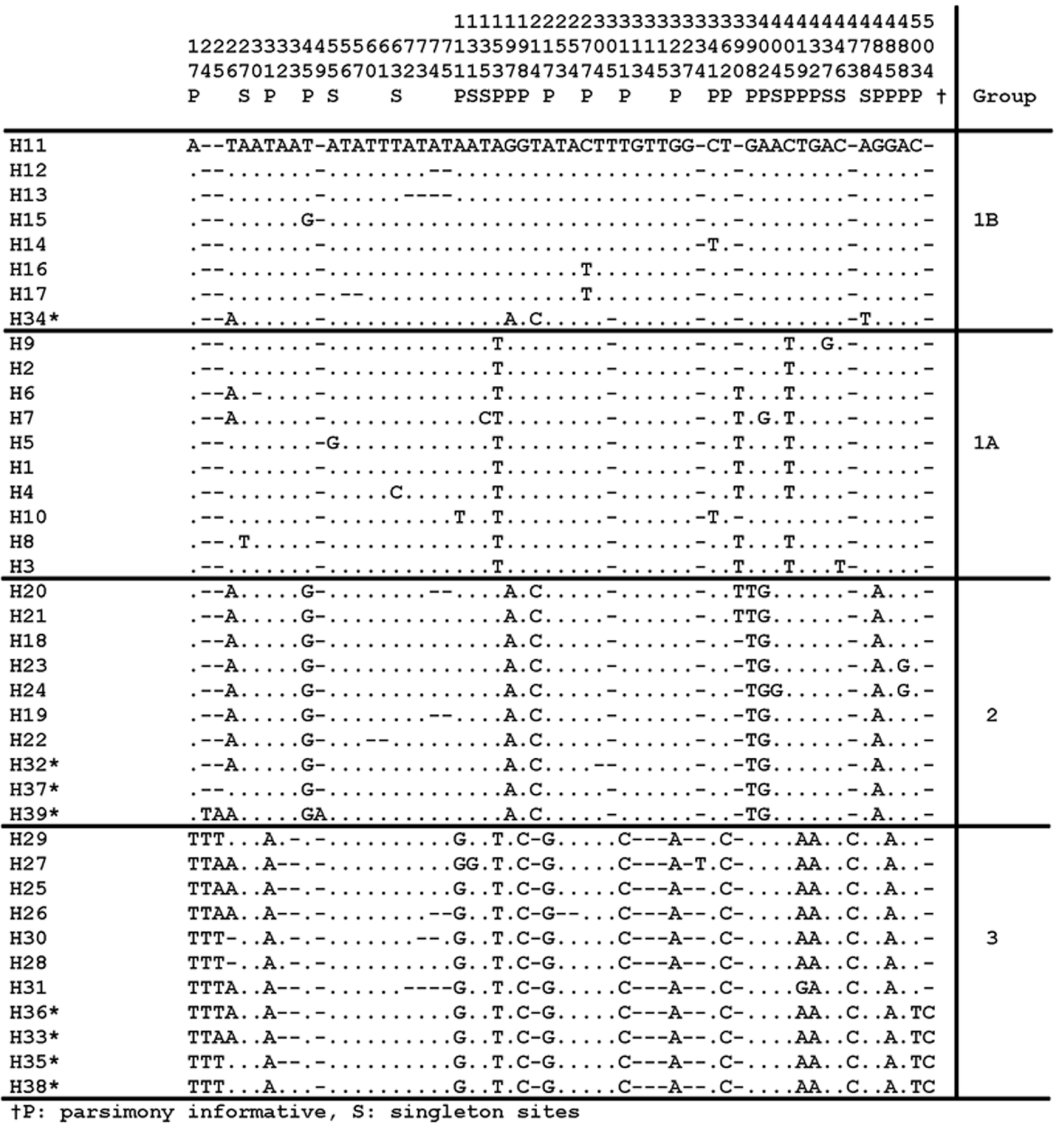
Haplotype sequence differences found in all 190 *Triatoma dimidiata* ITS2 sequences (39 haplotypes). Haplotypes are separated into the 4 ITS2 groups described in the text. Nucleotide positions are given (read vertically) at the top based on Clustal W alignment. “.” = identity, †S-singleton sites, P-parsimony informative sites, *newly identified haplotypes.

**Figure 2:**
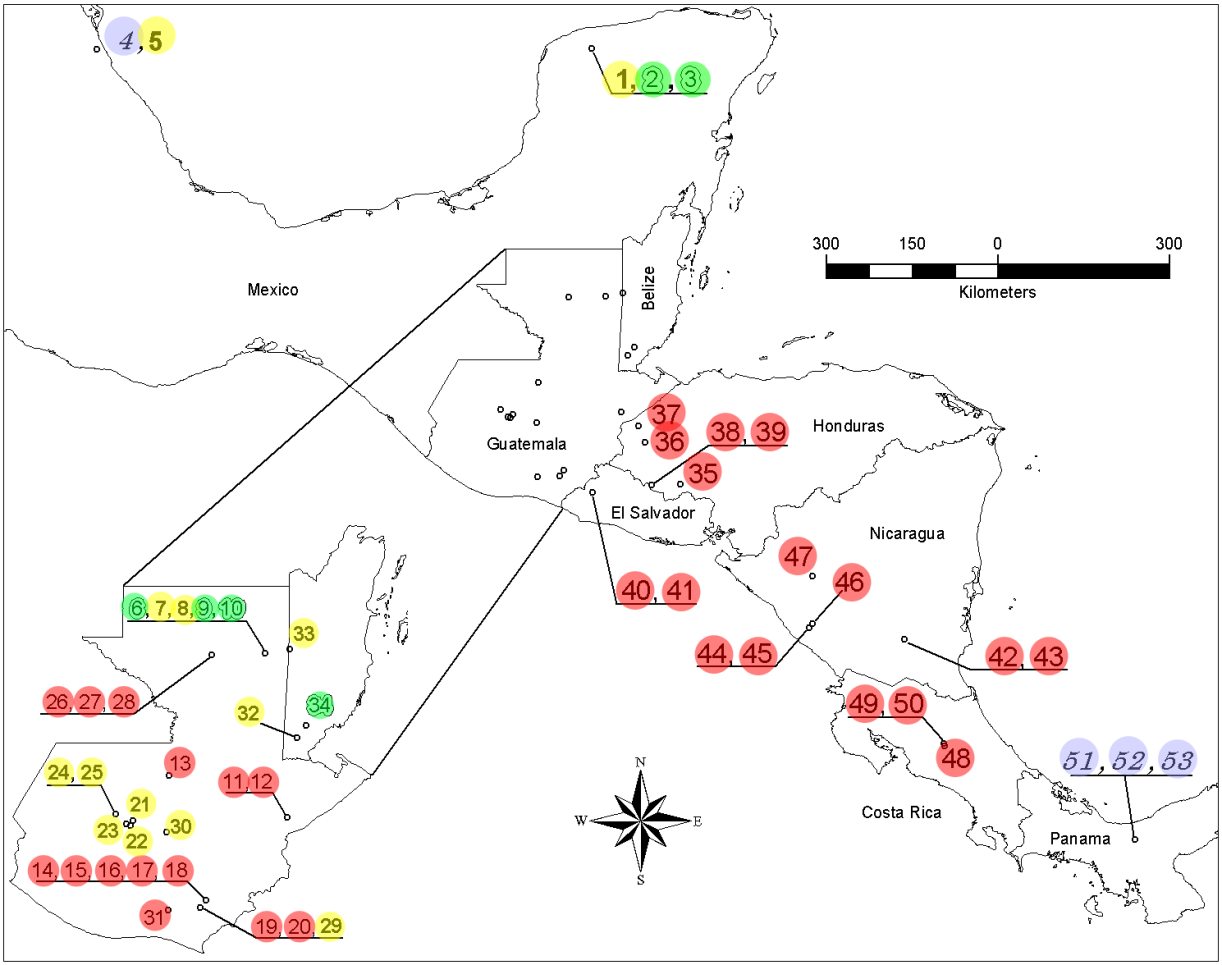
Map showing location of 53 *Triatoma dimidiata* isolates collected (see Table 1 for details). ITS2 groups: Group 1A (red), Group 1B (blue), Group 2 (yellow), Group 3 (green). Numbers identify individuals (Table 1).

**Figure 3:**
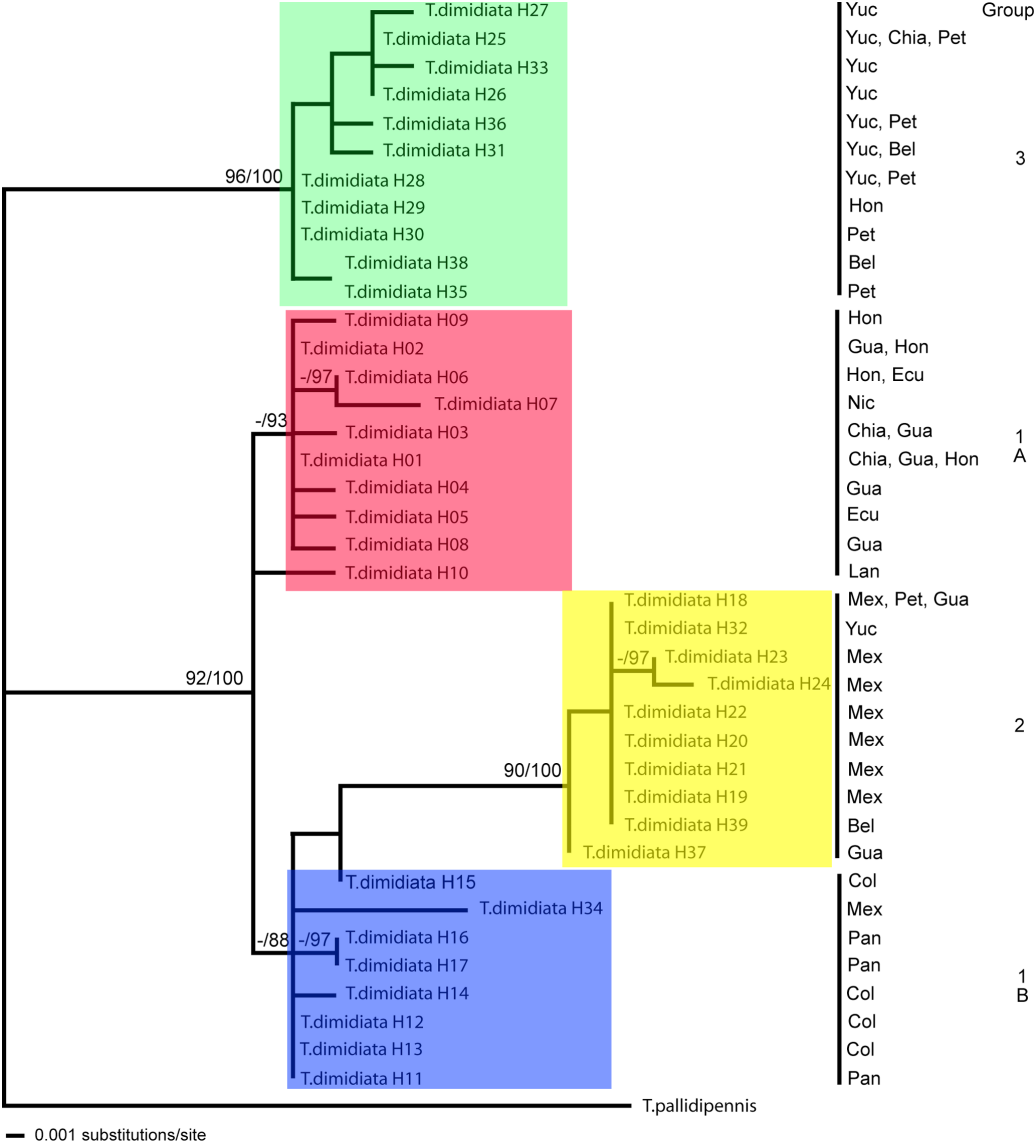
Phylogenetic maximum likelihood tree of all 39 haplotypes of *T. dimidiata* ITS2 sequence. Number of substitutions per site is indicated by the bar and bootstrap values higher than 75% / Bayesian posterior probabilities larger than 85% are indicated at the nodes. Yuc = Yucatan, Mexico (MX); Chiapas = Chiapas, MX; Peten = Peten, Guatemala (GT); Bel = Belize; Hon = Honduras; Ecu = Ecuador; Nic = Nicaragua; Lanquin = Lanquin, Alta Verapaz, Guatemala; Mex = Mexico; Pan = Panama; Col = Colombia. *T. pallidipennis* is the outgroup. ITS2 Groups coded as follows: 1A (red), 1B (blue), 2 (yellow), 3 (green).

We found 15 ITS2 haplotypes in the 53 sequences and compared these to the 31 available full-length ITS2 haplotypes (from 137 specimens) in GenBank (Fig 1). (The remaining 24 sequences in GenBank are truncated therefore haplotypes cannot be assigned and they are excluded from the analysis). Seven were among the 31 *T. dimidiata* haplotypes previously identified [38] and eight are unique (found in Belize, Guatemala and Mexico) to give a total of 39 haplotypes. Our phylogenetic and AMOVA analyses support the four distinct groups previously identified [38]: 1A (a Central American cluster, which we now extend into Costa Rica), 1B (Panama and Colombia [also includes one southern Mexico sample]), 2 (a southern Mexico cluster, which we now extend into southern Guatemala), and 3 (a quite distinct taxon found in Yucatan, Mexico Peten, Guatemala, and Cayo, BZ) (Figs 2 & 3). The complex microsatellite repeat around nucleotides ∼44–72 (5′-TT(AT)_5_TTT(AT)_7_-3′), shows SNPs and indels among individuals, however changes do not correlate with particular groups. There is a Group 3 signature sequence present around nucleotides ∼307–320 of 5′-CTGTATAAAACAAT-3′. The following four SNPs distinguish Group 2 individuals: a T to C transition at position 213, a G to T transversion at position 400, an A to G transition at position 404, and an A to G transition at position 485.

### Phylogenetic analysis based on ITS2

Separate MP, ML and Bayesian analysis of the ITS2 datasets did not generate discordant topologies among them. ML phylogenetic analysis shows the four groups with strong bootstrap support for each node and Group 2 derived from Group 1B (Fig 3). Results of the median-joining network analysis using ITS2 data show the same four groupings as the ML phylogram (data not shown) with Groups 1A and 1B the closest and central, Group 2 a bit more distant from those two and Group 3 the furthest. Interestingly, one of the newly reported haplotypes, H34, from Veracruz, MX, is in an intermediate position between Groups 1 and 2, which may represent a transitional state between the two or hybridization and further concerted evolution. Although it clusters with Group 1B on the ML tree (Fig 3), the 5′ end of the haplotype appears most closely related to Group 2 (Fig 1). Haplotype H10, representing the Lanquin, GT cave population, clusters with, but is the most distinct from the Central American group, 1A (Fig 3).

### Average nucleotide substitutions per site in ITS2

The average evolutionary distance within and between groups, calculated by the Kimura 2-parameter model, shows less than 0.5% divergence within each group (0.1–0.4%, diagonal, Table 3) with Group 1A (Central America) showing the greatest intragroup divergence (0.43%). Among groups, 1A and 1B are the most closely related (0.8% substitution), with Group 2 showing 2–2.5 times greater distance with groups 1A and 1B than between the latter two. Group 3 shows the greatest divergence from the three other groups, ∼3–4% which, by the molecular clock, translates into a time of 1.6–5.2 mya since the last common ancestor.

A hierarchical partitioning of variance shows that by far, most of the variance (90.4%, Table 4) can be accounted for by differences among the groups, which is highly significant (p<0.001) and supports the four-group classification [38]. The variance component among countries within groups and among individuals within countries is also significant, although with smaller values (Table 4).

**Table 4:** Hierarchical analysis of molecular variance for ITS2 in *T. dimidiata* populations.

Source of variation	d.f.	Sum of squares	Variance components	Percentage of variation
Among groups	3	222.45	6.81*** Va	90.2
Among countries within groups	9	14.06	0.317** Vb	4.2
Among individuals within countries	40	16.93	0.423*** Vc	5.6
Total	52	253.43	7.55100	

Groups: according to Median-joining Network Analysis, 1A, 1B, 2 & 3.

***: p<0.001; **: p<0.01.

The largest of the fixation indices was nearly one (F_ST_ = 0.944), indicating nearly completely distinct haplotypes among countries within groups. F_CT_ is also nearly equal to one (F_CT_ = 0.902) showing the significant variance among groups. These two indices show the importance of the groups, which explains most of the observed variance between haplotypes. F_CS_ indicates the importance of the variance between countries compared to the variances among and within countries (F_CS_ = 0.428). This F_CS_ shows that the variance among countries is slightly less than the variance among haplotypes within countries. The high amount of heterogeneity within countries could mean some populations within a country are isolated from others.

### Geographic distribution

Our ITS2 results support a cluster of “southern Mexican isolates” (Group 2, which we show extends into Guatemala) distinct from a Central American (Group 1A, overlapping in Guatemala) (Fig 2). Our data extends the range of Group 2 considerably southwards to include Quiche and Baja Verapaz, Guatemala, and also Belize, identifying overlap of Groups 1A and 2 in Guatemala. The remainder of Group 1A haplotypes are found in Central America (and our data extends this group southwards into Costa Rica), and 1B found in Panama and Colombia, with the exception of one Veracruz, Mexico haplotype (H34) that clusters with this latter group by ML phylogenetic analysis; however, the long length of the branch indicates it is diverged from Group 1B.

Unlike the geographically localized Groups 1A, 1B and 2, the more divergent taxon, Group 3, shows a scattered distribution, occurring along with Group 2 in Peten, Guatemala and Yucatan, Mexico (Fig 2). We identified four new haplotypes in this group and extend its distribution to Cayo, Belize. In contrast to reports associating distinct groups with specific localities and habitats [39], our data show several distinct groups are in sympatry, sometimes within the same city (Merida, MX) or even microhabitat (palm trees) in the same archeological site (Yaxhá, GT).

### Comparison with mt *cyt b* sequences

**Figure 4:**
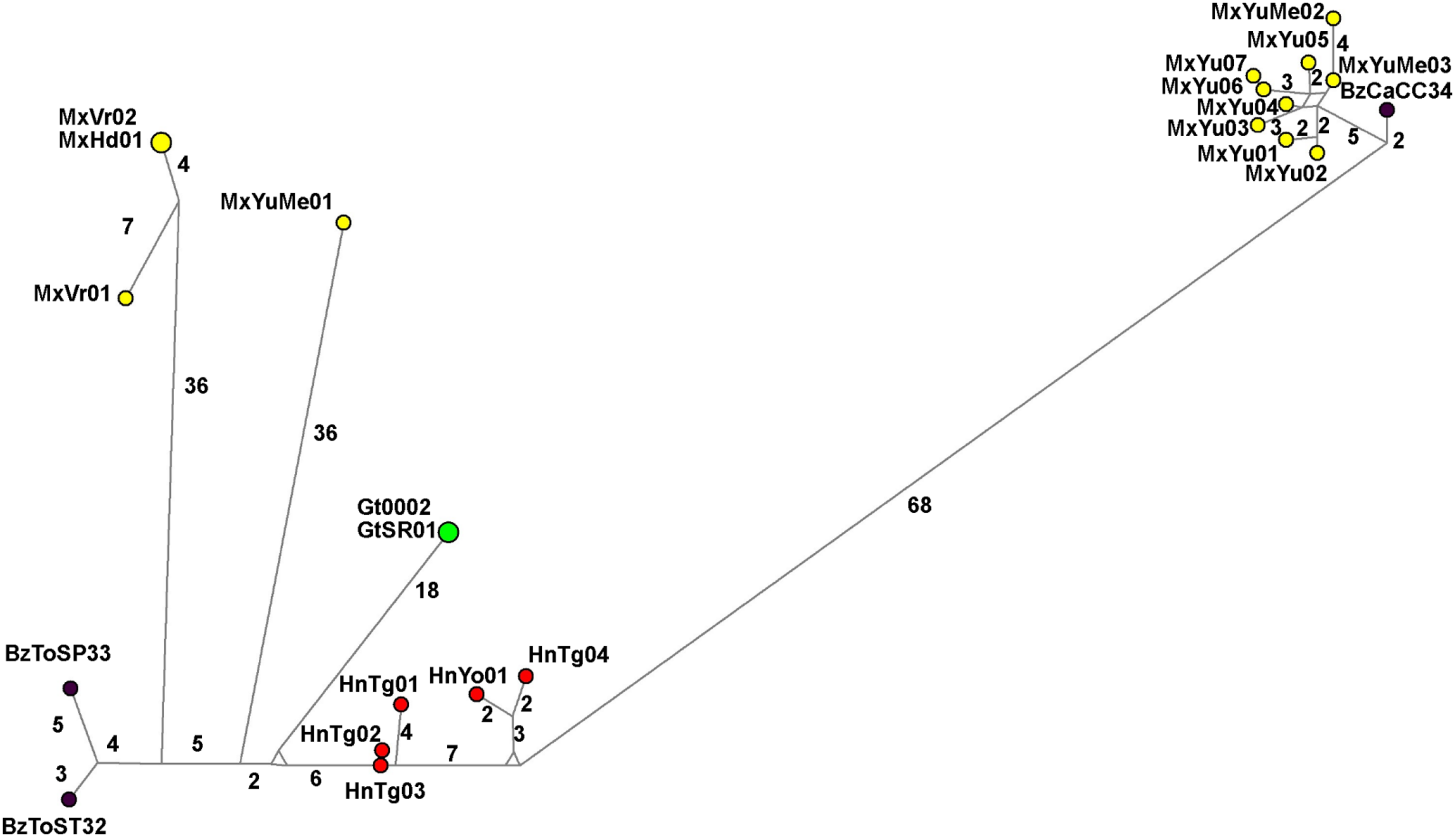
Network analysis of all 22 haplotypes of *T. dimidiata* mt *cyt b*. The size of the circle indicates how many individuals shared that haplotype. The colors indicate the country of the sample: Mexico-yellow, Honduras-red, Guatemala-green, Belize-black.

To check if Group 3 truly represents a distinct taxon in sympatry with other taxa we compared the ITS2 phylogenies with mt *cyt b* phylogenies from the six samples for which we had DNA available, representing ITS2 Groups 2 and 3 from Yucatan, Mexico and Belize. Those from ITS2 group 3 (MxYuMe02, MxYuMe03 and BzCCCa34 clearly fall into a distinct taxon also with mt *cyt b* sequence data, clustering with other isolates from Yucatan, Mexico, where other Group 3 individuals have been identified. As seen with ITS2, the distance is nearly as large as the distance from a different species, *T. pallidipennis*, used as an outgroup. In addition, network analysis shows a tight clustering, even closer than ITS2, quite separate from all others (Fig 4).

**Figure 5:**
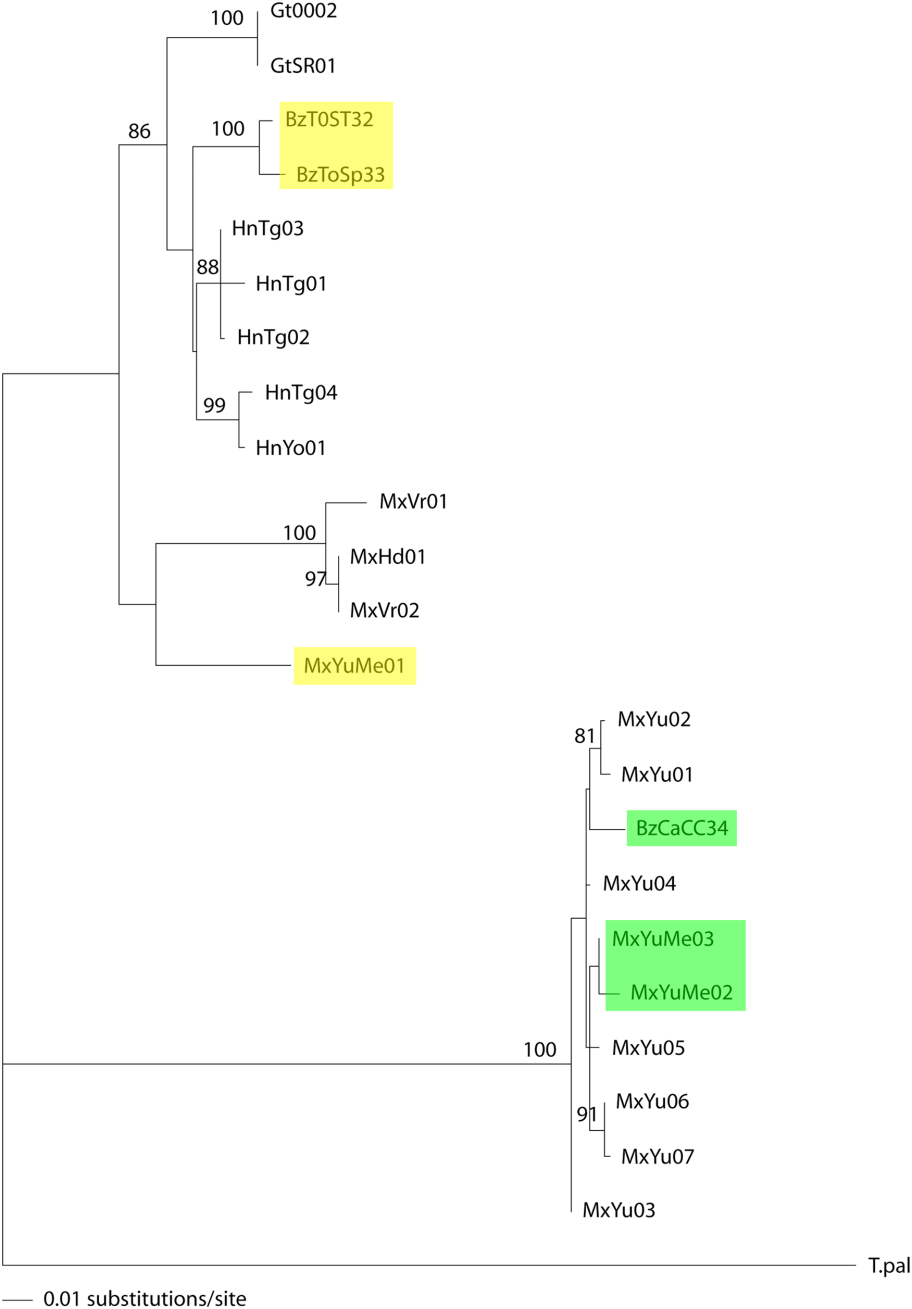
Phylogenetic maximum likelihood tree of all 23 distinct mt *cyt b* sequences of *T. dimidiata*. Number of substitutions per site is indicated by the bar and bootstrap values higher than 75% are indicated at the nodes. See Table 2 for sample ID. *T. pallidipennis* is the outgroup. ITS2 Group 2 is shown in yellow and Group 3 in green.

Interestingly, what are clearly distinct taxa as ITS2 Groups 1A and 2 (1B may not be represented in our mt *cyt b* data as we do not have samples from Panama and Colombia) are unresolved by mt *cyt b* data (<75% bootstrap value, Fig 5). Both the ML and network analysis show more of a clinal variation than distinct groups (Figs 4 and 5). So it appears that the distinct taxon (Group 3 by ITS2) is well supported, both by ITS2 and mt *cyt b*. However, the groupings of 1 and 2 are less clear with mt *cyt b* than ITS2. Southern Mexico and Central America isolates are more similar with mt *cyt b* than ITS2. More samples and markers will be needed to resolve this issue.

## Discussion

The sizes of the ITS2 sequences ranged from a small of 489 bp (in Group 3) to 499 bp (in Group 2), the latter only slightly larger than previously published *T. dimidiata* sequences (up to 497 bp) [38]. In addition, the AT bias is evident (75–76%) and is similar to that found in *Panstrongylus* species (75–79%) [29] and other Triatomini (77%) and Rhodniini (76%) [27].

The previously recognized complex microsatellite ((AT)_5_TTT(AT)_7_) was also present in the majority of all the haplotypes (69%), and in all groups, but interestingly is longer than that identified in other members of the phyllosoma which are (AT)_4_TTT(AT)_5–6_) [35], thus providing further evidence that *T. dimidiata* may be diverging from the phyllosoma complex. Our data also shows SNPs present within the microsatellite sequence which may limit the use of this sequence for assigning individuals to groups as has been recently proposed [35]. SNPs in ITS2 that are outside the microsatellite sequence were identified that are diagnostic for Groups 2 and 3. The Group 3 signature can be used to directly identify this group by PCR amplification without the need for sequencing (Dumonteil, et al., unpublished data). So ITS2 can be used to distinguish groups although the region containing the microsatellite sequence may not be the most useful portion.

### Taxonomy of *T. dimidiata*

Since its discovery, the species *T. dimidiata*, has been split and merged many times (reviewed in Dorn, et al. [11]). At the population level, genetic characters show that among domestic populations, geographically close *T. dimidiata* are similar [42] whereas generally the geographically more distant populations are diverged [59]; results expected if ancestral populations became separated. Until recently, the divergence was considered to be “clinal in nature” and “not segregated into clearly separable allopatric populations” [14]. However, recently taxonomists have suggested that *T. dimidiata* is more aptly considered a species complex [15] and rather than “clinal” differentiation, recent phenotypic and genetic data suggest *T. dimidiata* may be divided into distinct taxa.

Results using particular phenotypic or genetic markers divide *T. dimidiata* into two (antenna sensilla, [16] and male genitalia (Monroy, et al., unpublished data), three (head morphometry [17] and cytogenetics [19]) or four (cuticular hydrocarbon patterns [18]) distinct taxa. However, the divisions among these taxa do not always agree among different markers (e.g. different “cytotypes” are found in Peten, Guatemala and Yucatan, Mexico however, isolates from these two regions are grouped together [and different from all other isolates] by cuticular hydrocarbon patterns). Often there is an outlier population, but this is sometimes identified in Yucatan, Mexico, and/or Peten, Guatemala (by cuticular hydrocarbons, cytogenetics, male genitalia) or the Lanquin caves in Alta Verapaz, Guatemala or Boavita, Colombia (by head morphometry). To see if different markers are identifying the same or different taxa, it will be necessary to analyze a suite of markers on the same individuals.

Recently 31 haplotypes of ITS2 sequence from *T. dimidiata* across its geographic range grouped into four taxa by phylogenetic analysis [38]. Our ITS2 data from an additional eight haplotypes supports this classification and we show that the “southern Mexico” Group 2 extends well into southern Guatemala where it overlaps with the Central American Group 1A. This division between a southern Mexican (and Guatemalan) and Central American *T. dimidiata* (ITS2 estimated time of divergence of 1.02–2.47 my) is also supported by antenna structure, head morphometry, and cuticular hydrocarbon analysis. (Cytogenetics and morphometry of male genitalia do not resolve these two groups). However, these two groups are more closely clustered with mt *cyt b* data than is seen with ITS2 and more of a clinal variation is seen (Figs 4 & 5). Before we support the proposal to reassign the subspecies designations, *T. dimidiata maculipennis*, to the ITS2 southern Mexican group 2, and *T. dimidiata dimidiata*, to the ITS2 Central American taxon, Group 1A, it will be important to look at additional gene sequences and to use multiple markers, phenotypic and genotypic, all on the same samples.

Our data extends the reach of ITS2 Group 1A as far south as Costa Rica. With very few isolates of Group 1B examined so far, and most of these from Colombia, the division between Subgroups 1A and 1B by ITS2 is unclear and needs examination of additional samples in Panama and further north in Central America. In addition, subgroups 1A and 1B show the least intergroup divergence and additional samples, studied with multiple markers, will be needed to see if this is a true division or the “clinal” variation noted by Lent and Wygodzinsky [14]. Indeed, the topology of our ML tree using ITS2 differs somewhat from that published by Bargues, et al. [38] as Group 2 is derived from 1B in our tree and 1A in theirs.

Group 3, the putative cryptic species (*T.* spp. aff. *dimidiata*, [38]) is confirmed by our ITS2 data and mt *cyt b* sequence data, 100% bootstrap support, clearly separate from all other *T. dimidiata*. It appears to be widespread as we find it in Peten, Guatemala; Yucatan, Mexico, and Belize and it was previously shown to be as far north as Chiapas, MX and as far south as Yoro, Honduras [38]. Interestingly, we have clearly shown that Group 3 exists in sympatry with Group 2 in Peten, Guatemala and Yucatan, Mexico; more isolates are needed to see if this holds true for Belize as well. Finding the distinct groups in the same city or microhabitat in the same archeological site suggests that geographic separation is not essential for reproductive isolation. In addition, we see no association with habitat and group as sylvan and domestic samples are found in Groups 1A, 2 and 3 (we have incomplete habitat information for samples published by Bargues, et al. to assess Group 1B). Cross-breeding experiments are ongoing (Monroy, et al.) to begin to understand the mechanism of this reproductive isolation.

### Implications for control

A large body of literature shows that one of the major Chagas vectors in Mesoamerica, *T. dimidiata*, varies enormously in genetic, phenotypic traits and behaviorally across its geographic range (reviewed in [11]). The studies described here using ITS2 as well as mt *cyt b* here show a clear separation of the putative cryptic species. However groupings of the remaining populations seem to differ between these two markers. Clearly, information from more genes is needed to clearly understand the division among *T. dimidiata* taxa. Distinct taxa have significance for the epidemiology of the disease, e.g. in different localities where *T. dimidiata* is the only Chagas vector, the seropositivity rate in humans differs dramatically, e.g. from 0–18.5% in regions in Guatemala [60]. Distinct taxa may also affect control outcomes. Since 1997, the Central America Initiative for the Control of Chagas disease has shown dramatically different results following insecticide spraying in houses, e.g. in Nicaragua, the bugs did not return [61]; in stark contrast to rapid reinfestation in Jutiapa, Guatemala [62]. It is important to understand how much of the differences in epidemiology and control outcomes are due to distinct taxa of *T. dimidiata*. The area of Peten, Guatemala has not been included in the control program since most populations are sylvan. Deforestation and increasing encroachment of human populations in the area means that *T. dimidiata* could become domesticated in this region. It is critical to realize that there are at least two distinct *T. dimidiata* populations in this area (and in Mexico and Belize) as control measures are designed. This work has begun to clarify the taxonomic status of *T. dimidiata* from different geographic regions. For effective control it will be imperative to understand the mechanisms maintaining this reproductive isolation and the epidemiological importance of distinct taxa.

## Supporting Information

Alternative Language Abstract S1 Translation of the Abstract into French by Claudia Calderon
(0.05 MB PDF)

Alternative Language Abstract S2 Translation of the Abstract into Spanish by Claudia Calderon
(0.05 MB PDF)
