# Supplementary material for: Two Distinct Triatoma dimidiata (Latreille, 1811) Taxa Are Found in Sympatry in Guatemala and Mexico
Source: PLoS Negl Trop Dis. 2009 Mar 10;3(3):e393. doi: 10.1371/journal.pntd.0000393 (PMC2648038; doi:10.1371/journal.pntd.0000393)
Supplement: Alternative Language Abstract S1 — Translation of the Abstract into French by Claudia Calderon (0.05 MB PDF) [file pntd.0000393.s001.pdf]

Environ 10 millions de personnes sont infectées par *Trypanosoma cruzi*, l'agent impliqué dans la maladie parasitique la plus grave des Amériques, la maladie de Chagas. La grande majorité a acquis le parasite par la contamination avec les résidus du vecteur, le triatome. La transmission de Chagas a été largement réduite en Amérique du Sud, particulièrement dans les régions avec des vecteurs qui sont en grande partie domestiques. Cependant en Méso-Amérique, *Triatoma dimidiata*, un des vecteurs principaux de la maladie de Chagas, pose des défis spéciaux pour son contrôle, en partie dû à sa grande diversité au long de son étendue géographique, et à la recolonisation des maisons après traitement avec pesticides par les populations péridomestiques et sylvestres. Des observations récentes suggèrent que *T. dimidiata* fait partie d'un complexe d'espèces, qui peut inclure des espèces cryptiques. Cette ambiguïté taxonomique rend difficile le contrôle de la maladie. La séquence nucléaire de l'espaceur interne transcrit 2 (ITS2) de l'ADN ribosomal et celle du cytochrome b mitochondrial (mt *cyt b*) ont été employées pour analyser la taxonomie de *T. dimidiata* du sud du Mexique à l'Amérique Centrale. Les séquences d' ITS2 divisent *T. dimidiata* en quatre taxons. Les trois premiers se trouvent dans des régions géographiques spécifiques avec quelque chevauchement: (1) le sud du Mexique et le Nord du Guatemala (Groupe 2); (2) le Guatemala, le Honduras, El Salvador, le Nicaragua et le Costa Rica (Groupe 1A) ; et (3) le Panama (Groupe 1B). Nous étendons le Groupe 1A d'ITS2 jusqu'au Costa Rica, le Groupe 2 jusqu'au sud du Guatemala et nous apportons les premières informations sur le Belize, en identifiant les Groupes 2 et 3 dans ce pays. Le quatrième taxon, une espèce cryptique potentielle (Groupe 3), est dispersé dans diverses parties du Mexique, du Guatemala et du Belize. Nous montrons qu'il existe en sympatrie avec d'autres groupes présents au Petén (Guatemala) et au Yucatan (Mexique). Les données obtenues avec le mt *cyt b* confirment que cette espèce cryptique putative se trouve en sympatrie avec d'autres populations. Cependant alors que ITS2 montre une distinction claire des groupes restants, ceux-ci ne sont pas différenciés par le mt *cyt b*. Ce travail permet une meilleure compréhension de la taxonomie et de la division des populations de *T. dimidiata*, qui sont essentielles pour l'élaboration de stratégies de contrôle efficaces.
