# Supplementary material for: Two Distinct Triatoma dimidiata (Latreille, 1811) Taxa Are Found in Sympatry in Guatemala and Mexico
Source: PLoS Negl Trop Dis. 2009 Mar 10;3(3):e393. doi: 10.1371/journal.pntd.0000393 (PMC2648038; doi:10.1371/journal.pntd.0000393)
Supplement: Alternative Language Abstract S2 — Translation of the Abstract into Spanish by Claudia Calderon (0.05 MB PDF) [file pntd.0000393.s002.pdf]

Aproximadamente 10 millones de personas están infectadas con *Trypanosoma cruzi*, agente causal del mal de Chagas, una de las enfermedades parasíticas más serias del continente americano. La gran mayoría adquiere el parásito por contaminación a través de vectores triatominos. La transmisión de la enfermedad se ha reducido drásticamente en Sud América, especialmente en áreas en donde el vector es principalmente doméstico. Sin embargo, *Triatoma dimidiata*, uno de los principales vectores en Mesoamérica, representa un reto especial para el control debido a su diversidad a lo largo de su rango de distribución geográfica y a la rápida recolonización de las casas después de la aplicación de pesticidas a partir de poblaciones peridomésticas y silvestres. Datos recientes sugieren el que *T. dimidiata* sea un complejo de especies, que puede incluir especies crípticas. Esta ambigüedad taxonómica ha dificultado el control de dicha enfermedad. La secuencia nuclear del segundo espaciador transcrito interno (ITS2) del ADN ribosomal y la del gen mitocondrial citocromo b (mt *cit b*) fueron utilizadas para analizar la taxonomía de *T. dimidiata*, desde el sur de Mexico hasta el istmo centroamericano. La secuencia de ITS2 divide a *T. dimidiata* en cuatro taxones. Los primeros tres se encuentran en regiones geográficas especificas que pueden solaparse: (1) el sur de México y Guatemala (Grupo 2); (2) Guatemala, Honduras, El Salvador, Nicaragua y Costa Rica (Grupo 1A); y (3) Panamá (Grupo 1B). Extendemos el Grupo 1A del ITS2 hasta Costa Rica, el Grupo 2 hasta Guatemala y mostramos las primeras informaciones sobre muestras de Belice, identificando en ese país los Grupos 2 y 3. El cuarto taxón, lo constituye lo que podría ser una especie críptica (Grupo 3), que se distribuye en áreas de Guatemala, Mexico y Belice. Mostramos que existe en simpatría con otros grupos de Petén (Guatemala) y Yucatán (México). Los datos del mt *cit b*, un marcador mitocondrial, confirman los resultados antes mencionados. Sin embargo, a diferencia de la clara distinción de los grupos restantes obtenida por ITS2, éstos no se separan con el mt *cit b*. Este trabajo contribuye a la comprensión de la taxonomía y estructura poblacional de *T. dimidiata*, que son esenciales para el diseño de estrategias de control efectivas.
